# Analysis of immortal-time effect in post-infarction ventricular septal defect

**DOI:** 10.3389/fcvm.2023.1270608

**Published:** 2023-10-19

**Authors:** Héctor González-Pacheco, Jorge Arturo Ortega-Hernandez, Jesús Ángel Meza-López, Luis Alejandro Soliz-Uriona, Daniel Manzur-Sandoval, Rodrigo Gopar-Nieto, Diego Araiza-Garaygordobil, Daniel Sierra-Lara, Eduardo Arias-Sánchez, Juan Pablo Sandoval, Alfredo Altamirano-Castillo, Salvador Mendoza-García, Arturo Arzate-Ramírez, Francisco Martin Baranda-Tovar, Humberto Martinez, Álvaro Montañez-Orozco, Luis Augusto Baeza-Herrera, Alejandro Sierra-González De Cossio, Alexandra Arias-Mendoza

**Affiliations:** ^1^ Coronary Care Unit, National Institute of Cardiology, Mexico City, Mexico; ^2^ Department of Interventional Cardiology, National Institute of Cardiology, Mexico City, Mexico; ^3^ Department of Cardiovascular Critical Care Unit, National Institute of Cardiology, Mexico City, Mexico; ^4^ Department of Cardiovascular Surgery, National Institute of Cardiology, Mexico City, Mexico

**Keywords:** post-infarction ventricular septal defect, ST-elevation myocardial infarction, acute myocardial infarction, immortal times bias, transcatheter closure, surgical closure, conservative management

## Abstract

**Introduction:**

Time-fixed analyses have traditionally been utilized to examine outcomes in post-infarction ventricular septal defect (VSD). The aims of this study were to: (1) analyze the relationship between VSD closure/non-closure and mortality; (2) assess the presence of immortal-time bias.

**Material and methods:**

In this retrospective cohort study, patients with ST-elevation myocardial infarction (STEMI) complicated by VSD. Time-fixed and time-dependent Cox regression methodologies were employed.

**Results:**

The study included 80 patients: surgical closure (*n* = 26), transcatheter closure (*n* = 20), or conservative management alone (*n* = 34). At presentation, patients without VSD closure exhibited high-risk clinical characteristics, had the shortest median time intervals from STEMI onset to VSD development (4.0, 4.0, and 2.0 days, respectively; *P* = 0.03) and from STEMI symptom onset to hospital arrival (6.0, 5.0, and 0.8 days, respectively; *P* < 0.0001). The median time from STEMI onset to closure was 22.0 days (*P* = 0.14). In-hospital mortality rate was higher among patients who did not undergo defect closure (50%, 35%, and 88.2%, respectively; *P* < 0.0001). Closure of the defect using a fixed-time method was associated with lower in-hospital mortality (HR = 0.13, 95% CI 0.05–0.31, *P* < 0.0001, and HR 0.13, 95% CI 0.04–0.36, *P* < 0.0001, for surgery and transcatheter closure, respectively). However, when employing a time-varying method, this association was not observed (HR = 0.95, 95% CI 0.45–1.98, *P* = 0.90, and HR 0.88, 95% CI 0.41–1.87, *P* = 0.74, for surgery and transcatheter closure, respectively). These findings suggest the presence of an immortal-time bias.

**Conclusions:**

This study highlights that using a fixed-time analytic approach in post-infarction VSD can result in immortal-time bias. Researchers should consider employing time-dependent methodologies.

## Graphical Abstract

The first part of the figure explains the natural history of post-myocardial infarction ventricular septal defect (VSD), where the first event is the AMI itself and the 2nd the rupture and creation of a VSD this first time until the VSD closure is where most of the mortality is concentrated precluding that an intervention can be performed (median to the closure of 22 days). The second part of the current study sheds light on where a time-fixed analysis does include the immortal time bias giving the closure either surgical or transcatheter and advantage in the hazard ratios, but when counting this bias in a Cox time-variable analysis where all the patients start in a medical group and then mathematically rearrange in their respective interventions this survival advantage is lost.

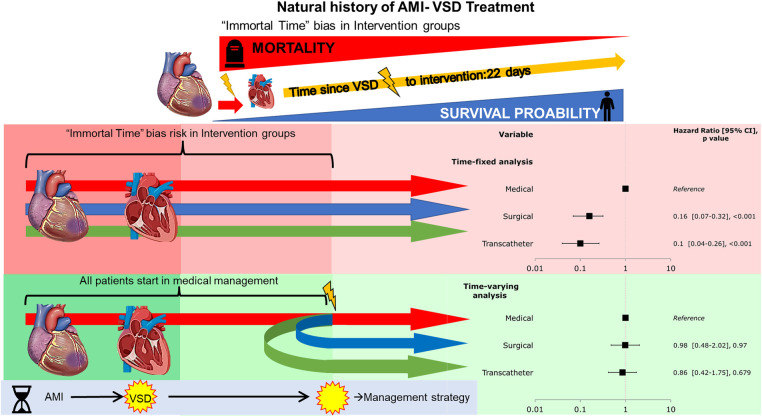

## Introduction

A post-infarction ventricular septal defect (VSD) is a rare but potentially life-threatening complication of acute myocardial infarction (AMI) (1). In the pre-reperfusion era, the incidence in patients with AMI was 1%–3% (2), but the advent of reperfusion therapy saw a significant decrease in the incidence of VSD to 0.2%–0.5% (3). This complication has a bimodal presentation, with a high incidence in the first day and 3–5 days after AMI (1). The mortality rate of this complication compared with AMI without VSD remains very high, with in-hospital mortality rates of about 45% for surgically treated patients and 90% for those treated medically (4). It is generally associated with hemodynamic instability and/or cardiogenic shock, and it is associated with an extremely poor prognosis, with an 87% mortality in AMI-VSD compared with 59% in the SHOCK trial for patients without VSD (5). Thus, surgical closure is the current standard for treating patients with post-AMI VSD (6). However, surgical intervention is often delayed allowing initial healing for at least 2 weeks until patients are more stable, and the friable myocardial tissue has healed (7, 8). To stabilize high-risk patients or to treat post-surgical residual VSD, percutaneous transcatheter defect closure and adjunct to surgical closure of post-AMI VSD have become alternatives (9).

This study aimed to investigate the prevalence of post-AMI VSD, patients' characteristics, predictors, and outcomes by analyzing immortal time bias and prove the survivor treatment selection bias in a tertiary care center for cardiovascular disease.

## Materials and methods

We performed a retrospective cohort study from a referral cardiovascular specialty center, the Coronary Care Unit database of the National Institute of Cardiology in Mexico City. The study cohort comprised patients admitted to our Coronary Care Unit diagnosed with acute ST-segment elevation myocardial infarction (STEMI) from January 2006 to December 2022, complicated with VSD. The diagnosis of STEMI was based on the universal definition of myocardial infarct (10). We gathered demographic characteristics, medical history, physiological parameters at admission (i.e., blood pressure and heart rate), biochemical findings, in-hospital treatments, and in-hospital mortality. Baseline creatinine clearance was estimated using the Cockcroft–Gault formula. The diagnosis and characterization of VSD were obtained by two-dimensional echocardiographic imaging with color flow Doppler, cardiac catheterization, and computerized tomography. The size and site of the rupture, grade shunt, and amount of injury in the right and left ventricles were determined.

Two types of VSD were defined: simple and complex. Simple ruptures have a straight-simple connection between the left and right ventricles, happening at the same level in both chambers without gross hemorrhage or tear. A complex rupture is an interventricular communication with a tortuous route, with a tract that might spread into areas far from the AMI site and with hemorrhage and disruption of myocardial tissue (11).

Cardiogenic shock was defined as systolic blood pressure <90 mmHg for ≥30 min or the need for catecholamines to maintain systolic blood pressure >90 mmHg, clinical pulmonary congestion, and organ hypoperfusion with any of the following symptoms: cold extremities; confusion or altered mental status; oliguria; or blood lactate >2.0 mmol/L (12). Right ventricular dysfunction or failure was defined for echocardiographic metrics, including fractional area change (FAC), tricuspid annular plane systolic excursion (TAPSE), and a systolic S' wave, using the tissue Doppler technique. TAPSE <17 mm, FAC <35%, and S' <9.5 cm/s were considered pathologic values. Coronary artery disease was defined as >50% stenosis in one of the three major epicardial coronary arteries.

The primary endpoint of this study was all-cause in-hospital mortality, defined as death from any cause during the same hospitalization. For our analysis, patients were subdivided into three groups according to treatment as follows: (i) surgical closure; (ii) percutaneous transcatheter closure, and (iii) conservative management alone.

## Statistical analysis

All categorical data were summarized as frequencies and percentages. Continuous variables are presented as median and 25th and 75th percentiles (interquartile ranges, IQRs) or mean ± SD, as appropriate. Statistically significant differences between groups were assessed, using either chi-square or Fisher's test for categorical variables. Continuous variables were analyzed using Shapiro–Wilk tests to assess the distribution and were compared using analysis of ANOVA for normally distributed variables or the Kruskal–Wallis test for non-normally distributed variables.

The primary study outcome was all-cause in-hospital mortality. In-hospital mortality rates were calculated for each group of patients according to the treatment received and expressed as a percentage; chi-square tests evaluated group differences. Additionally, using the cohort of patients admitted during the same period without post-infarction VSD as a reference, Cox proportional hazards regression was used to assess the effect of VSD closure as a time-fixed exposure on in-hospital mortality. Survival was plotted with the Kaplan–Meier curve, and a log-rank test assessed group differences. A multivariate Cox's proportional hazards regression model was used to adjust for the potential confounding based on the established associations between treatment received and in-hospital mortality. The candidate covariates were those associated with mortality in a univariate analysis, which included all the baseline characteristics, clinical features on presentation, characteristics of the ventricular septal defect, the different times analyzed, and VSD closures that had *P* < 0.05, as well as those recognized as prognostic factors based on previous medical knowledge.

Finally, because there was evidence of a substantial effect of immortal time bias/survivor treatment selection bias in the closure group, time to closure was used as a time-dependent covariate. For the time-dependent Cox proportional hazard regression analysis, we used the COUNTING PROCESS method in PROC PHREG to handle time-dependent covariates. The first step was to construct a dataset with multiple records per patient, with one record for each period during which all the covariates remained constant. We used the SAS code to construct this dataset. The second step was to use the special syntax in PROC PHREG to estimate the model. We used the baseline function in PROC PHREG to estimate the baseline hazard function and the: (start, stop) syntax to adjust for the study group. The time-to-event variable was the time from the start of each interval to the event's occurrence (death). The following variables were included in the final model: in the study group and the multivariate analysis, SBP <100 mmHg, cardiogenic shock at admission, estimated glomerular filtration rate (eGFR), and time from STEMI to emergency department arrival, and in the intervention-only population, early closure <14 days. We used the Wald chi-square test to assess the significance of the coefficients and the likelihood ratio test to assess the model's overall fit. We also performed a sensitivity analysis by excluding patients with missing data on any covariates.

Results were reported using two-tailed significance; statistical significance was set at *P* < 0.05. All tests were two-sided, and *P* < 0.05 was considered statistically significant. IBM SPSS Statistics for Windows, version 23 (IBM Corp., Armonk, NY, USA), and SAS for Academics were used.

## Results

During the analyzed period, between January 2006 to December 2022, 8,022 patients were admitted with a diagnosis of STEMI. Of them, 80 (1%) had a VSD complication, of whom 26 (32.5%), 20 (25%), and 34 (42.5%) patients underwent surgical closure of the VSD, percutaneous transcatheter closure, and conservative management without performing closure of the VSD, respectively. The baseline and admission characteristics of patients in the three groups are summarized in Table 1. The mean age was 64 ± 4 years, with no differences between groups. Most of the patients were men (71.6%); however, there was a greater number of women (45%) in the patients treated by percutaneous transcatheter closure. Among the whole sample, there were high rates of a history of diabetes mellitus (48.8%) and hypertension (57.5%), and the prevalence of other cardiovascular risk factors, such as obesity or smoking, as well as cardiovascular diseases, showed no significant difference between groups.

**Table 1:** Baseline characteristics of patients.

	Overall (*n* = 80)	Surgery closure (*n* = 26)	Percutaneous transcatheter closure (*n* = 20)	Conservative management (*n* = 34)	*P-*value
Age, mean (SD), (years)	64.4 ± 7.9	62.4 ± 7.3	64.0 ± 9.2	66.0 ± 7.3	0.20
Men, *n* (%)	63 (78.8)	25 (96.2)	11 (55.0)	27 (79.4)	0.003
Body mass index, mean (SD), (kg/m^2^)	26.0 ± 3.6	25.6 ± 3.1	26.6 ± 4.1	26.7 ± 3.7	0.14
Medical history					
Current smoking, *n* (%)	22 (27.5)	8 (30.8)	4 (20.0)	10 (29.4)	0.68
Hypertension, *n* (%)	46 (57.5)	13 (50.0)	12 (60.0)	21 (61.8)	0.63
Dyslipidemia, *n* (%)	13 (16.3)	2 (7.7)	6 (30.0)	5 (14.7)	0.12
Diabetes, *n* (%)	39 (48.8)	13 (50.0)	10 (50.0)	16 (47.1)	0.96
Previous MI, *n* (%)	5 (6.3)	1 (3.8)	1 (5.0)	3 (8.8)	0.70
Previous PCI, *n* (%)	2 (2.5)	1 (3.8)	0 (0.0)	1 (2.9)	0.69
Previous heart failure, *n* (%)	6 (7.5)	2 (7.7)	2 (10.0)	2 (5.9)	0.85
Previous stroke, *n* (%)	2 (2.5)	1 (3.8)	0 (0.0)	1 (2.9)	0.69

MI, myocardial infarction; PCI, percutaneous coronary intervention.

At presentation, managed patients without VSD closure compared with those with VSD closure were more likely to have high-risk clinical features, including lower systolic blood pressure, higher heart rate, higher Killip class, lower left ventricular ejection fraction, and right ventricular dysfunction. Among the whole sample, 21 (26.3%) patients presented with cardiogenic shock (CS) at admission; however, the higher frequency of CS in the group of patients managed without VSD closure should be highlighted (11.5%, 10.0%, and 47.1% for surgical closure, percutaneous transcatheter closure and conservative management groups, respectively; *P* = 0.004). Patients with conservative management had higher glycaemia levels, and laboratory values demonstrated significant end-organ dysfunction, as evidenced by lactic acidosis, renal impairment, and elevated liver transaminases (Table 2).

**Table 2:** Clinical features, laboratory data and echocardiographic findings at hospital admission of patients.

	Overall (*n* = 80)	Surgery closure (*n* = 26)	Percutaneous transcatheter closure (*n* = 20)	Conservative management (*n* = 34)	*P-*value
Location of infarct:					
Anterior wall, *n* (%)	42 (52.5)	7 (26.9)	17 (85.0)	18 (52.9)	<0.0001
Inferior/posterior wall, *n* (%)	38 (47.5)	19 (73.1)	3 (15.0)	16 (47.1)	
Systolic blood pressure, mean (SD) (mmHg)	105 ± 20	112 ± 18	110 ± 21	98 ± 19	0.01
Mean arterial pressure, mean (SD) (mmHg)	80 ± 15	85 ± 15	83 ± 14	73 ± 13	0.004
Heart rate, mean (SD) (beats/min)	94 ± 23	90 ± 23	94 ± 17	96 ± 27	0.46
Cardiogenic shock at admission, *n* (%)	21 (26.3)	3 (11.5)	2 (10.0)	16 (47.1)	0.001
Left ventricular ejection fraction, median, (IQR) (%)	40 (33–50)	45 (37–52)	43 (40–52)	35 (30–48)	0.03
Right ventricular dysfunction*, *n* (%)	46 (57.5)	17 (65.4)	11 (55.0)	18 (52.9)	0.60
Glomerular filtration rates, median, (IQR) (ml/min)	51 (36–76)	55 (41–83)	63 (46–76)	44 (30–67)	0.11
Blood glucose level, median, (IQR) (mg/dl)	160 (117–179)	127 (98–183)	158 (128–216)	226 (143–343)	0.003
Alanine aminotransferase, median, (IQR), (U/L)	60 (28–285)	54 (23–120)	51 (18–79)	113 (37–1,193)	0.01
Aspartate aminotransferase, median, (IQR), (U/L	99 (29–304)	50 (25–200)	38 (22–118)	239 (80–758)	0.001
Arterial pH, median (IQR)	7.41 (7.35–7.46)	7.43 (7.39–7.46)	7.41 (7.37–7.47)	7.40 (7.30–7.45)	0.09
Bicarbonate, median, (IQR) (mEq/L)	19 (16–22)	20 (18–22)	20 (18–23)	17 (14–20)	0.01
Lactate, median, (IQR) (mmol/L)	1.8 (1.2–3.4)	1.4 (1.2–2.6)	1.4 (1.0–2.2)	2.9 (1.6–6.6)	0.003

*Determined by echocardiogram measures: TAPSE <17 mm, Fractional area change <35%, and S' <9.5 cm/s.

Anterior wall location of the infarction was most frequent in 42 patients (52.5%) and inferior or posterior in 38 patients (47.5%); six patients had an extension of the infarction to the right ventricle. However, of the 42 patients with anterior infarction, 17 (40.5%) underwent percutaneous transcatheter closure and only 7 (16.7%) surgical closure; the remaining 18 (42.8%) patients did not undergo VSD closure. In contrast, of the 38 patients with inferior/posterior infarction, 19 (50%) underwent surgical closure, only three (7.9%) percutaneous transcatheter closure, and 16 (42.1%) patients received only conservative management.

Of the 80 patients, only 20 (25%) received reperfusion therapy; most received thrombolytic therapy in other hospitals and were transferred to our institution. Only seven (8.8%) patients received reperfusion therapy with primary percutaneous coronary intervention. Coronary angiography was performed in 65 (81.3%) patients, and about half had multivessel disease, and non-primary percutaneous coronary intervention was undertaken in only nine (11.3%) patients; there was no significant difference between the groups. Moreover, the use of inotropic agents, vasopressors, intra-aortic balloon pumps, and invasive ventilation was higher in patients with conservative management than in patients who underwent VSD closure (Table 3).

**Table 3:** In-hospital management and procedures in patients with AMI.

	Overall (*n* = 80)	Surgery closure (*n* = 27)	Percutaneous transcatheter closure (*n* = 20)	Conservative management (*n* = 33)	*P-*value
Reperfusion therapy					
No reperfusion, *n* (%)	60 (75.0)	22 (81.5)	17 (85.0)	21 (63.6)	0.04
Primary PCI, *n* (%)	7 (8.8)	0 (0.0)	0 (0.0)	7 (21.2)	
Thrombolysis in our institution, *n* (%)	1 (1.3)	1 (3.7)	0 (0.0)	0 (0.0)	
Thrombolysis in another hospital, *n* (%)	12 (15.0)	4 (14.8)	3 (15.0)	5 (15.2)	
Coronary angiography, *n* (%)	65 (81.3)	26 (96.3)	17 (85.0)	22 (66.7)	0.01
Number of diseased vessels. (*n* = 65)					
1-vessel disease	31 (47.7)	10 (38.5)	11 (64.7)	10 (45.5)	0.29
2-vessel disease	21 (32.3)	8 (30.8)	5 (29.4)	8 (36.4)	
3-vessel disease	13 (20.0)	8 (30.8)	1 (5.9)	4 (18.2)	
Left main artery disease	4 /6.2)	2 (7.7)	0 (0.0)	2 (9.1)	0.46
Non-primary PCI, *n* (%)	9 (11.3)	3 (11.1)	3 (15.0)	3 (9.1)	0.80
Inotropes IV, *n* (%)	51 (63.8)	12 (44.4)	13 (65.0)	26 (78.8)	0.02
Vasopressors IV, *n* (%)	52 (65.0)	12 (44.4)	14 (70.0)	26 (78.8)	0.01
IABP, *n* (%)	58 (72.5)	20 (74.1)	13 (65.0)	25 (75.8)	0.67
Mechanical ventilation, *n* (%)	42 (52.5)	8 (29.6)	9 (45.0)	25 (75.8)	0.001

PCI, percutaneous coronary intervention, IABP, intra-aortic balloon pump.

### Characteristics of ventricular septal defects

Overall, in most patients (*n* = 51, 63.8%), the location of VSDs was apical, 33.8% (*n* = 27) inferior/basal, and only 2.5% (*n* = 2) anterior. On the other hand, 60% had complex characteristics, and 23.8% were simple; the remaining 16.3% could not be classified because of a lack of information. Overall, the size of the VSD had a median of 12.5 (IQR, 7.2–21.5) mm; patients with surgical closure were more likely to have a larger VSD; in contrast, patients without closure were more likely to have smaller defects, but with no significant differences between groups [13.5 (IQR, 8.2–26.7), 13.0 (IQR, 10.0–16.0), and 10.0 (IQR, 5.5–21.5) mm for surgical closure, percutaneous transcatheter closure, and conservative management groups, respectively; *P* = 0.57]. Other mechanical complications (papillary muscle rupture, free wall rupture, and aneurysm/pseudoaneurysm) were documented in 32 (40%) patients, and aneurysms/pseudoaneurysms were the most frequent (Table 4).

**Table 4:** Characteristics of the ventricular septal defect.

	Overall (*n* = 80)	Surgery closure (*n* = 26)	Percutaneous transcatheter closure (*n* = 20)	Conservative management (*n* = 34)	*P-*value
Location of the VSD					
Anterior/Apical, *n* (%)	53 (66.3)	14 (53.8)	18 (90.0)	21 (61.8)	0.02
Inferior/basal, *n* (%)	27 (33.8)	12 (46.2)	2 (10.0)	13 (38.2)	
Types of ventricular rupture					
Simple, *n* (%)	19 (23.8)	6 (22.2)	8 (40.0)	5 (15.2)	0.19
Complex, *n* (%)	48 (60.0)	18 (66.7)	10 (50.0)	20 (60.6)	
Not classifiable, *n* (%)	13 (16.3)	3 (11.1)	2 (10.0)	8 (24.2)	
VSD size, median, (IQR) (mm)	12.5 (7.2–21.5)	13.5 (8.2–26.7)	13.0 (10.0–16.0)	10.0 (5.5–25.7)	0.57
Mechanical complication associated	32 (40.0)	16 (61.5)	10 (50.0)	6 (17.6)	0.002
Mitral papillary muscle dysfunction, *n* (%)	4 (12.5)	1 (6.3)	2 (20.0)	1 (16.7)	
Free wall rupture, *n* (%)	4 (12.5)	2 (12.5)	2 (20.0)	0 (0.0)	
Aneurysm/Pseudoaneurysm, *n* (%)	24 (75.0)	13 (81.3)	6 (60.0)	5 (83.3)	

VSD, ventricular septal defect.

### Analysis of the different times involved

**Figure 1:**
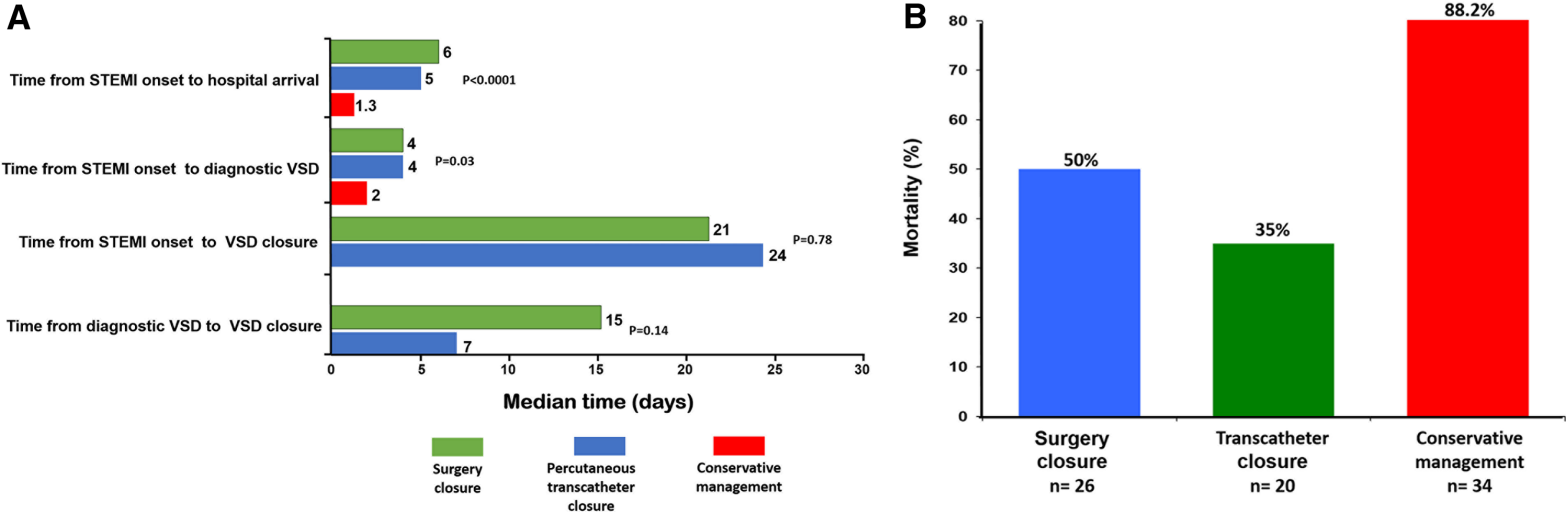
(**A**) Analysis of the different times involved. (**B**) Groups of post-infarction VSD patients according to treatment with corresponding in-hospital mortality rate.

Figure 1A shows the different times analyzed and compared between the patient groups. Among the whole sample, the STEMI symptom onset times to our hospital arrival had a median of 4.0 (IQR, 0.6–8.0) days; patients with conservative management had shorter STEMI symptom onset times compared with those with VSD closure [6.0 (IQR, 3.7–8.2), 5.0 (IQR, 2.5–16), and 1.3 (IQR, 0.1–3.8) days for surgical closure, percutaneous transcatheter closure, and conservative management groups, respectively; *P* < 0.0001].

Among the whole sample, the median time from onset of STEMI to development of VSD was 3.0 (IQR, 1.0–8.0) days, whereas patients with conservative management had a shorter time compared with those with VSD closure [4.0 (IQR, 2.0–9.0), 4.0 (IQR, 2.0–20.0), and 2.0 (IQR, 1.0–4.0) days for surgical closure, percutaneous transcatheter closure, and conservative management groups, respectively; *P* = 0.03].

The median time from onset of STEMI to surgical or percutaneous closure of VSD was 22.0 (IQR, 12.0–45.0) days, and there was no difference between the groups [21.0 (IQR, 13.0–38.0) vs. 24.0 (IQR, 8.0–to 52.0) days for surgical closure and percutaneous transcatheter closure groups, respectively; *P* = 0.14]. Likewise, the median time between VSD detection to repair was [14 (IQR, 5.0–27.0) days (15.0 (IQR, 10.0–29.0), and 7.0 (IQR, 3.0–25.0) days for surgical closure and percutaneous transcatheter closure groups, respectively; *P* = 0.78].

### Predictors of in-hospital mortality (time-fixed analysis)

**Figure 2:**
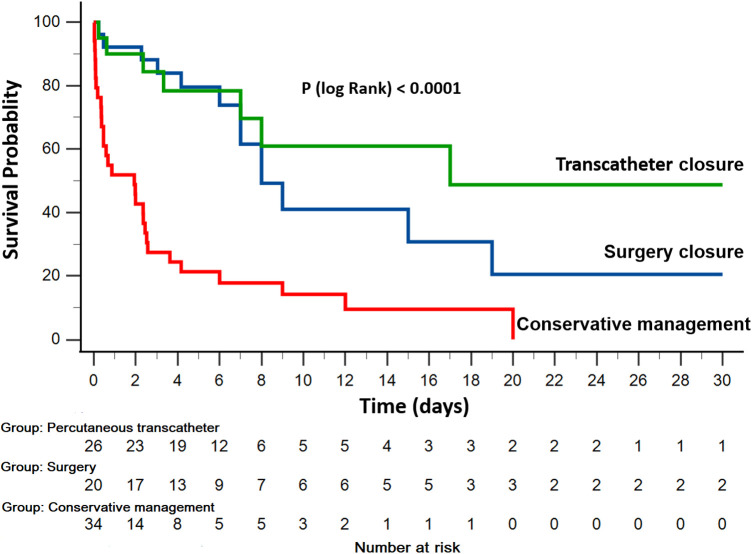
Kaplan-Meir curves for in-hospital survival by management.

In the global cohort, in-hospital mortality occurred in 50 patients (62.5%). The unadjusted in-hospital mortality rate was significantly higher among patients who did not undergo defect closure (50%, 35%, and 88.2% for surgery closure, percutaneous transcatheter closure, and conservative management groups, respectively; *P* < 0.0001; Figure 1B). Using the patient group without post-AMI VSD as a reference in the Cox proportional hazards model, patients admitted with post-AMI VSD who did not undergo repair of the defect showed a very high in-hospital mortality risk [hazard ratio (HR) 6.0, 95% CI 3.51–10.55, *P* < 0.0001; HR 3.5, 95% CI 1.69–7.56, *P* = 0.001; and HR 30.9, 95% CI 21.41–44.62, *P* < 0.0001, for surgery closure, percutaneous transcatheter closure, and conservative management groups, respectively; Figure 2].

In the cohort of patients who underwent VSD closure, the patients receiving treatment of VSD closure within 14 days of STEMI the risk ratio of mortality was 3.30-fold increase (95% CI 1.32–8.25, *P* = 0.01) compared with those treated after 14 days.

An adjusted multivariate Cox proportional hazards regression model was made with all statistically significant univariate predictors of in-hospital mortality at admission. The analysis demonstrated that eGFR of 30–60 ml/min and performing VSD closure within 14 days from STEMI onset (phase acute) were associated with a 3.20-fold and 3.90-fold increase in the risk of in-hospital mortality, respectively. Additionally, the closure of the defect by any of the methods (HR 0.13, 95% CI 0.05–0.31, *P* < 0.0001; and HR 0.13, 95% CI 0.04–0.36, *P* < 0.0001, for surgery closure and percutaneous transcatheter closure groups, respectively) and as well as the greater delay in time from STEMI onset to hospital arrival (HR 0.64, 95% CI 0.48–0.86, *P* = 0.003) were associated with lower in-hospital mortality (Table 5).

**Table 5:** Multivariate Cox analysis for the prediction of in-hospital mortality.

	Univariate Cox analysis			Multivariate Cox analysis		
	Hazard ratio	95% Confidence interval	*P*-value	Hazard ratio	95% Confidence interval	*P*-value
Systolic blood pressure <100 mmHg	2.89	1.62–5.14	<0.0001	1.03	0.47–2.23	0.93
Cardiogenic shock at admission	3.85	2.12–6.98	<0.0001	1.66	0.77–3.54	0.19
Glomerular filtration rates >60 ml/min	Reference group					
Glomerular filtration rates 30–60 ml/min	2.97	1.49–5.9	0.002	3.20	1.43–7.11	0.004
Glomerular filtration rates <30 ml/min	3.35	1.48–7.54	0.001	2.30	0.88–6.00	0.08
Time from AMI onset to hospital arrival (per 4 days)	0.64	0.49–0.83	0.001	0.64	0.48–0.86	0.003
Time from AMI onset to closure of VSD <14 days^a^	3.30	1.32–8.25	0.01	3.90	1.33–11.44	0.01
Without closure of VSD	Reference group					
Surgery closure of VSD	0.16	0.07–0.32	<0.0001	0.13	0.05–0.31	<0.0001
Percutaneous transcatheter closure of VSD	0.10	0.04–0.26	<0.0001	0.13	0.04–0.36	<0.0001

AMI, acute myocardial infarction; VSD, ventricular septal defect.

^a^ The analysis was performed only for the intervention groups: percutaneous and surgical.

### Proving a survival bias in the intervention of post infarction VSD patients

A logistic regression from STEMI onset time to closure of the VSD (surgical or interventional) or death demonstrated a time-dependent gradient where it is more likely to get a VSD closure (*P* < 0.0001) with an OR of 1.15 (95% CI 1.08–1.23) per day since the AMI; this was corroborated with a spline that showed linearity *df *= 1, X2 = 13.2, *P* < 0.0001.

**Figure 3:**
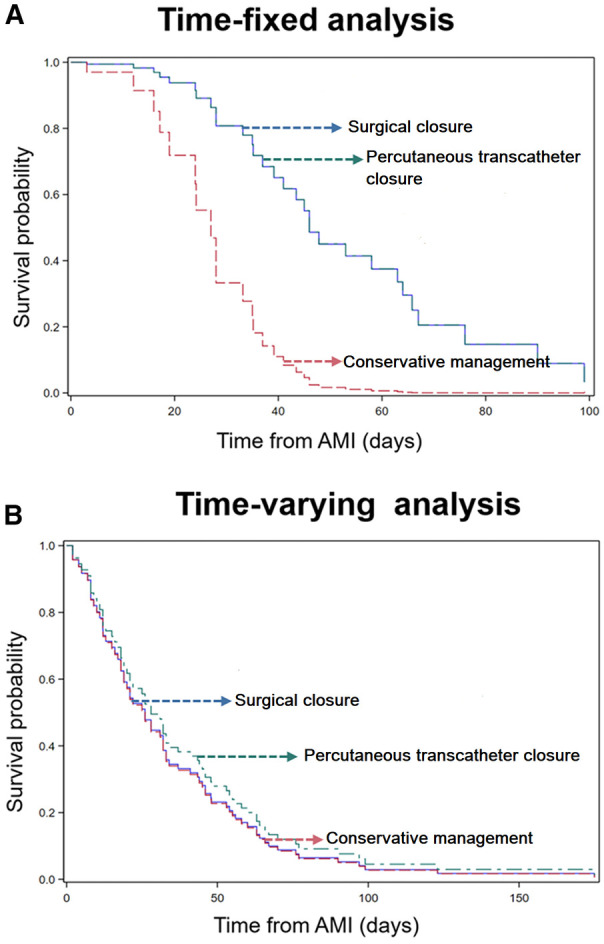
Kaplan–Meier curves assessing the impact of post-infarct VSD management on the probability of in-hospital mortality. (**A**) Demonstrates a significant association between VSD closure, analyzed as a time-fixed variable, and improved hospital survival. (**B**) Illustrates the Kaplan–Meier curves for VSD closure analyzed as a time-varying variable, with the red curve representing conventional management, the blue curve representing transcatheter closure, and the green curve representing surgical closure. The overlapping curves suggest that treatment with or without VSD closure, analyzed as a time-varying variable, does not show significant differences in in-hospital mortality.

When we undertook a Cox time-dependent variable analysis of the cohort of time of STEMI, it showed that although in the time-fixed variable a significance was seen, this was lost in this time-sensitive analysis (HR 0.95, 95% CI 0.45–1.98, *P* = 0.90; and HR 0.88, 95% CI 0.41–1.87, *P* = 0.74, for surgery closure and percutaneous transcatheter closure groups, respectively), thus proving a survival bias for intervention selection (Figure 3).

### Survival analysis from landmark event (STEMI)

We conducted a time-fixed analysis to investigate the effect of treatment on post-AMI VSD patients in terms of survival. We considered the time from STEMI to the event (death or discharge) and found significant differences for surgery closure (HR = 0.16, 95% CI 0.07–0.32, *P* < 0.0001) and percutaneous transcatheter closure (HR = 0.1, 95% CI 0.04–0.26, *P* < 0.0001). We also identified several significant variables associated with survival, including SBP <100 mmHg (HR = 2.89, 95% CI 1.62–5.14, *P* < 0.0001), CS at admission (HR = 3.85, 95% CI 2.12–6.98, *P* < 0.0001), eGFR 30–60 ml/min (HR = 2.97, 95% CI 1.49–5.9, *P* = 0.002), GFR <30 ml/min (HR = 3.35, 95% CI 1.48–7.54, *P* = 0.001), and time to arrival from STEMI (HR = 0.64, 95% CI 0.9–0.83, *P* = 0.001 for every 4 days). Furthermore, an early closure from STEMI was associated with an HR of 3.03-fold increase (95% CI 1.32–8.25, *P* = 0.01) (Table 6).

**Table 6:** Multivariate Cox analysis for the prediction of in-hospital mortality.

	Univariate Cox analysis			Multivariate Cox analysis		
	Hazard ratio	95% Confidence interval	*P*-value	Hazard ratio	95% Confidence interval	*P*-value
Systolic blood pressure <100 mmHg	2.89	1.62–5.14	<0.0001	1.03	0.47–2.23	0.93
Cardiogenic shock at admission	3.85	2.12–6.98	<0.0001	1.66	0.77–3.54	0.19
Glomerular filtration rates >60 ml/min	Reference group					
Glomerular filtration rates 30–60 ml/min	2.97	1.49–5.9	0.002	3.20	1.43–7.11	0.004
Glomerular filtration rates <30 ml/min	3.35	1.48–7.54	0.00	2.30	0.88–6.00	0.08
Time from AMI onset to hospital arrival (per 4 days)	0.64	0.49–0.83	0.001	0.64	0.48–0.86	0.003
Time from AMI onset to closure of VSD <14 days^a^	3.30	1.32–8.25	0.01	3.90	1.33–11.44	0.01
Without closure of VSD	Reference group					
Surgery closure of VSD	0.16	0.07–0.32	<0.0001	0.13	0.05–0.31	<0.0001
Transcatheter closure of VSD	0.10	0.04–0.26	<0.0001	0.13	0.04–0.36	<0.0001

AMI, acute myocardial infarction; VSD, ventricular septal defect.

^a^ The analysis was performed only for the intervention groups: percutaneous and surgical.

In the multivariate analysis, we lost significance for the presence of hypotension (HR = 1.03, 95% CI 0.47–2.23, *P* = 0.93) and for CS at admission (HR = 1.66, 95% CI 0.77–3.54, *P* = 0.19). However, eGFR 30–60 ml/min had an HR of 3.2-fold increase (95% CI 1.43–7.11, *P* = 0.004), whereas eGFR <30 ml/min lost significance (HR = 2.3, 95% CI 0.88–6.01, *P* = 0.08). Surgical or percutaneous closure was associated with increased survival (HR = 0.13, 95% CI 0.05–0.30, *P* < 0.0001, and HR = 0.13, 95% CI 0.04–0.36, *P* < 0.0001, respectively). When the time from STEMI to closure was considered, an early closure was associated with a HR of 3.9-fold increase (95% CI 1.33–11.44, *P* = 0.01) (Table 6).

We also conducted a Cox time-dependent variable analysis of the cohort of patients to assess the impact of the timing of the intervention on the survival outcome. This analysis revealed that although a significant difference was observed in the time-fixed variable analysis, this difference was lost in the time-sensitive analysis (X2 = 0.17, *P* = 0.916) with an HR of 0.98 (95% CI 0.48–2.02, *P* = 0.97) in surgical candidates and 0.86 (95% CI 0.42–1.75, *P* = 0.679) in patients who underwent intervention closure of VSD. This finding suggests the presence of a survival bias for intervention selection, as patients who underwent early intervention had a worse survival outcomes (Table 7).

**Table 7:** Time-varying Cox regression.

	Univariate Cox analysis			Multivariate Cox analysis		
	Hazard ratio	95% Confidence interval	*P*-value	Hazard ratio	95% Confidence interval	*P*-value
Systolic blood pressure <100 mmHg				0.84	0.4–2.06	0.71
Cardiogenic shock at admission				1.57	0.46–5.30	0.46
Glomerular filtration rates >60 ml/min	Reference group					
Glomerular filtration rates 30–60 ml/min				1.40	0.79–2.48	0.24
Glomerular filtration rates <30 ml/min				1.76	0.78–0.96	0.17
Time from AMI onset to closure of VSD <14 days^a^	2.30	1.31–4.03	0.03	4.21	1.89–9.5	<0.001
Without closure of VSD	Reference group					
Surgery closure of VSD	0.98	0.48–2.02	0.97	0.95	0.45–1.98	0.90
Transcatheter closure of VSD	0.86	0.42–1.75	0.67	0.88	0.41–1.87	0.74

AMI, acute myocardial infarction; VSD, ventricular septal defect.

^a^ The analysis was performed only for the intervention groups: percutaneous and surgical.

Even after adjustment for potential confounding factors, no significant differences were observed (X2 = 0.10, *P* = 0.947) in surgical candidates (HR = 0.95, 95% CI 0.45–1.98, *P* = 0.901) or patients who underwent percutaneous transcatheter closure (HR = 0.88, 95% CI 0.41–1.87, *P* = 0.744). Additionally, the time-fixed parameters, such as SBP <100 mmHg (HR 0.84, 95% CI 0.34–2.06, *P* = 0.713), eGFR with 30–60 ml/min HR = 1.40 (95% CI 0.79–2.48, *P* = 0.244), and <30 ml/min HR = 1.76 (95% CI 0.78–3.96, *P* = 0.171), and CS HR = 1.57 (95% CI 0.46–5.3, *P* = 0.463), were also lost. When we analyzed the time-variable parameters, acute closure <14 days still was associated with an HR of 4.21-fold increase (95% CI 1.89–9.35, *P* < 0.0001) (Table 7).

## Discussion

In this study, we aimed to evaluate the presence of immortal time bias in patients with post-AMI VSD and its potential impact on interpreting results obtained using fixed-time analysis methods. To address this, we employed a time-varying Cox regression to analyze survival outcomes.

Post-AMI VSD remains a challenging complication despite advancements in reperfusion therapies. Although the incidence has decreased, the mortality rate remains high. In our study population, we observed a higher post-AMI VSD prevalence than reported in the literature (1% vs. 0.27%) (13). This difference may be attributed, in part, to the limited availability of reperfusion therapy (75%) and the fact that our study was conducted at a single tertiary center.

Our research found high in-hospital mortality rates for patients with post-infarction VSD, consistent with previous observational studies. Medical management had the highest mortality rate (>90%), while surgical closure and percutaneous transcatheter closure showed lower rates (42%–61% and 32%–55%, respectively) (9, 14–16). These results align with earlier observations indicating a management selection bias and the confounding effects of survivorship bias, with delayed repair likely due to a more stable and fibrotic myocardium but also due to survival bias because the patients who are most likely to die early are removed from the analyses (16).

Although patients who received conservative management presented promptly between the onset of STEMI at admission to our hospital (less than 1 day) because of high-risk conditions, this likely influenced the fact that the patients did not receive the possibility of VSD closure. On the other hand, patients who underwent VSD closure procedures arrived at the hospital 5 days after the STEMI with better hemodynamic stability, allowing for closure later, at 22 days from the onset of the STEMI. In addition, our continuous analysis revealed that patients were more likely to receive treatment as time progressed.

The timing of VSD closure is known to significantly impact patient mortality, with better outcomes observed when the closure is performed after a certain period. This can be attributed to the evolution and healing of the infarcted myocardium, providing a more stable substrate for repair (16). Studies have consistently shown that a shorter time between rupture and surgery is associated with unfavorable outcomes, regardless of hemodynamic status, and that the precise timing of surgery depends on the patient's hemodynamic status (8). The Society of Thoracic Surgeons National Database showed that overall, in-hospital mortality was greater than 60% if surgery was performed within the first 24 h, 54.1% if the repair was within 7 days from AMI and decreased significantly to 18.4% if the repair was performed >7 days from AMI [mortality by surgical status: elective (13.2%) vs. emergent (56.0%) vs. salvage (80.5%)] (14). With percutaneous ventricular septal rupture closure, reports in the literature are similar, with mortality decreasing as patients progress from the acute to the chronic hin we found that VSD closure within 14 days (acute phase) was associated with a 2.57-fold increase in the risk of in-hospital mortality, a result that agrees with the findings of other studies, in which risk ratio of mortality for patients with acute vs. chronic post-AMI VSD closure showed a 3.34-fold increase (20).

Our study confirmed that patients managed conservatively without VSD closure had the highest mortality rates, highlighting the need to consider selection and time-to-AMI biases in future studies. Conversely, patients who underwent VSD closure demonstrated better survival rates, likely because of their improved clinical and hemodynamic stability and the partial healing of the myocardium post-infarction. In this context, Flynn et al. (21) conducted a meta-analysis of 25 studies of post-AMI VSD, highlighting the most significant risks of bias in confounding, selection bias, and selection of reported outcomes.

Diab et al. (20) recently commented on the importance of the immortal time bias effect in cardiovascular scenarios requiring surgical treatment. Our finding supports critical care researchers' recommendation that using a time-fixed analytic approach does not avoid the potential impact of immortal time bias on research in critically ill patients, suggesting that time-dependent analyses can be used to reduce its impact (22, 23). It is important to acknowledge the presence of immortal time bias in our study population. Immortal time refers to the period between the onset of AMI and the time of closure, surgery, or percutaneous intervention. This bias may lead to overestimating the impact of VSD closure on in-hospital mortality. Our time-dependent analyses revealed no significant differences in survival, emphasizing the critical role of time to AMI in the prognosis of this condition.

These findings have significant implications for managing and interpreting outcomes in patients with post-AMI VSD. As we continue to explore the impact of bias on reported outcomes, we must consider the timing of AMI events and their relationship to hemodynamic status and surgical risk. This study highlights the importance of understanding and addressing bias in future studies to improve the quality of evidence and, ultimately, the management of this devastating complication of AMI.

On the way forward, it will be important to establish the role of the distinct mechanical circulatory support devices and also consider the heart transplant as an early option in patients with very high-risk post-AMI VSD, for which, until now there is limited information and the application of time-dependent variable analysis, this could give a true value in the treatment of these patients (24, 25).

## Study limitations

Our study has several limitations. First, there was the inherent limitation of a retrospective analysis and the fact that it reflected the experiences of a single tertiary center specializing in cardiovascular diseases. Secondly, it should be noted that many patients did not receive early reperfusion therapy, which can influence myocardial tissue damage and subsequently affect the outcome of the patients. Lastly, the intensity of treatment, including but not limited to surgery, interventional, or mechanical circulatory support, could lead to different outcomes in our cohort.

## Conclusion

Our investigation represents the first attempt to elucidate the potential impact of immortal time bias in patients with post-AMI VSD. Utilizing a time-fixed analytic approach in this patient population may introduce immortal time bias, as patients who underwent VSD closure exhibited higher survival rates because of factors such as improved clinical and hemodynamic stability and partial healing of the vulnerable myocardium following the infarction. To address this bias, we employed a time-varying Cox regression model to analyze the immortal time bias. Our study highlights the importance of accounting for immortal time bias and adjusting for other relevant variables when assessing the survival benefit of post-AMI VSD closure on patient outcomes.

## Data Availability

The raw data supporting the conclusions of this article will be made available by the authors, without undue reservation.
